# Supplementary material for: lociPARSE: A Locality-aware Invariant Point Attention Model for Scoring RNA 3D Structures
Source: J Chem Inf Model. 2024 Nov 11;64(22):8655–64. doi: 10.1021/acs.jcim.4c01621 (PMC11600500; doi:10.1021/acs.jcim.4c01621)
Supplement: Supplementary file 1 — ci4c01621_si_001.pdf [file ci4c01621_si_001.pdf]

Supplementary Information for

lociPARSE: a locality-aware invariant point attention model for scoring RNA 3D structures

Sumit Tarafder[1] and Debswapna Bhattacharya[1*]

[1]Department of Computer Science, Virginia Tech, Blacksburg, Virginia, 24061, USA

Contents

1 Supplementary Tables **2**

1.1 Training dataset 2

1.2 Test datasets 2

1.2.1 Test30 and hyperparameter optimization set 2

1.2.2 CASP15 test set 3

2 Supplementary Figures **4**

2.1 Ability to rank the structures in terms of lDDT and RMSD 4

2.1.1 Top 1 distribution 4

2.1.2 Top 10 distribution 5

2.2 Training dataset distributions 6

2.3 Test dataset distributions 6

2.4 Interpretation of attention map 7

2.5 Distinguishability on test sets 8

*Corresponding Author. Email: dbhattacharya@vt.edu

1 Supplementary Tables

1.1 Training dataset

We generated 37 decoys for each of the 1,399 RNA targets using various deep-learning techniques, physics-based RNA folding methods, and PyRosetta perturbation methods. The total number of structures in our training set is 51,730. The training set was divided into two subsets: a training set and a validation set, with a split ratio of 80:20. The training set was used to train the architecture, while the validation set was used to save the model based on the best loss value which is used for evaluation throughout. The RNA 3D structural models and their corresponding ground truth lDDTs used to train and validate lociPARSE with a detailed description can be found here [1].

Table S1: List of the number of decoys per RNA target in our in-house training set

Method	Number of decoys
DeepFoldRNA	6
trRosettaRNA	10
RoseTTAFoldNA	1
DRfold	6
RhoFold	1
SimRNA	1
DeepFoldRNA + PyRosetta	12
Total	37

1.2 Test datasets

1.2.1 Test30 and hyperparameter optimization set

We generated 25 decoys for each target in Test30 and hyperparameter optimization dataset TS60. So the total number of structures in the Test30 set is 750. The decoy structures of CASP15 were collected from the CASP15 website which consists of all the submitted models in CASP15 by all groups. All the structural models for the three datasets can be found here [1].

Table S2: Number of decoys per RNA target in our in-house benchmark sets.

Method	Number of decoys per target
DeepFoldRNA	6
trRosettaRNA	10
RoseTTAFoldNA	1
DRfold	7
RhoFold	1
Total	25

1.2.2 CASP15 test set

Table S3: List of the number of decoys per RNA target in CASP15 test set

Target	Number of decoys per target
R1107	131
R1108	115
R1116	145
R1117	153
R1126	140
R1128	137
R1136	158
R1138	130
R1149	138
R1156	145
R1189	136
R1190	132
Total	1660

2 Supplementary Figures

2.1 Ability to rank the structures in terms of lDDT and RMSD

2.1.1 and 2.1.2 show the performance of all the quality assessment methods in terms of the ability to sort the pool of structures for each target in our 2 benchmark test sets Test30 and CASP15 following the analysis of top deep learning RNA quality assessment method in literature ARES [2].

2.1.1 Top 1 distribution

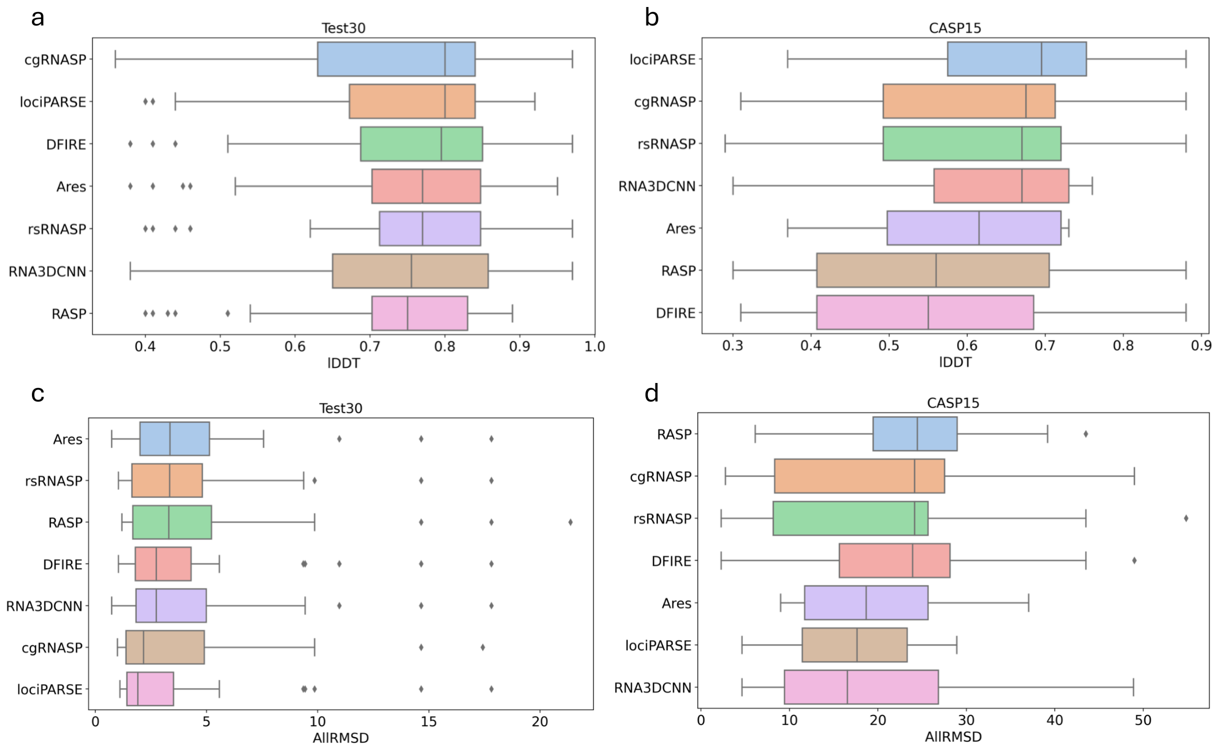

Figure S1: (a) and (b) represent the distribution of lDDT for targets in Test30 and CASP15 datasets respectively in terms of the top-ranked structure for each target assessed by 7 different methods sorted in descending order of median values. (c) and (d) shows the corresponding distribution of all-atom RMSD for the same analysis.

2.1.2 Top 10 distribution

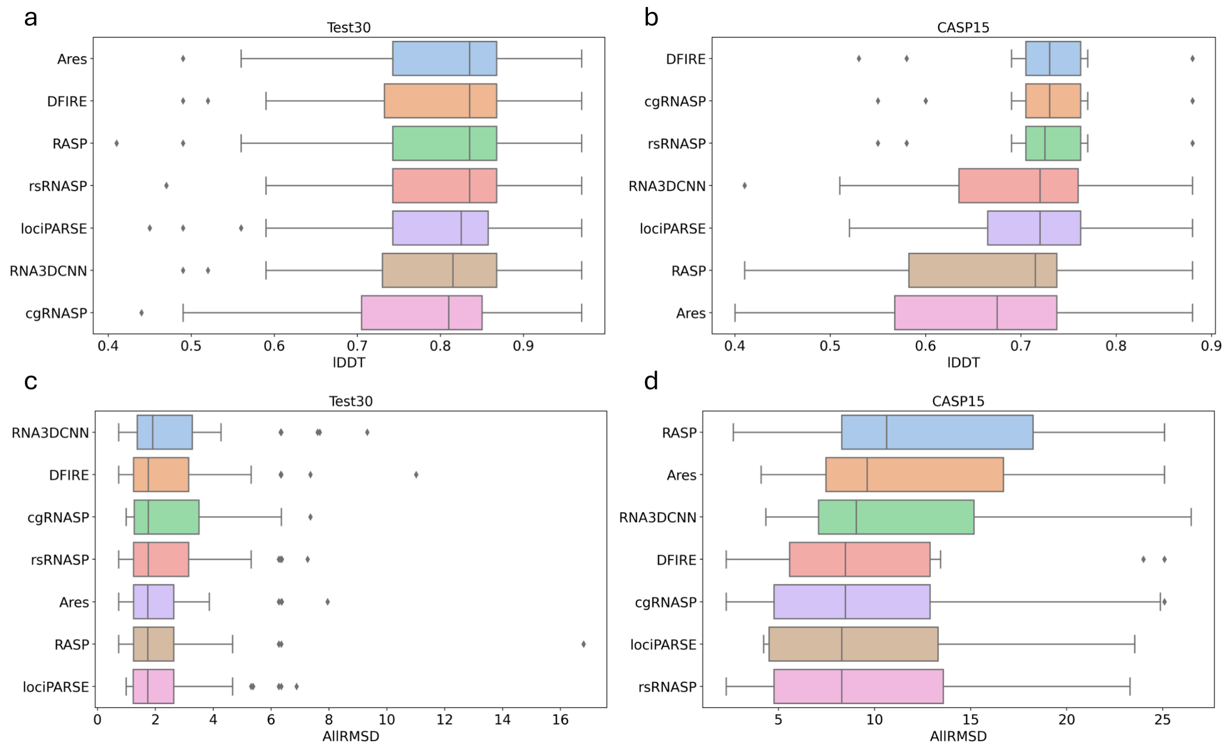

Figure S2: (a) and (b) represent the distribution of lDDT for targets in Test30 and CASP15 datasets respectively in terms of the highest lDDT among 10 best-scoring structural models for each target assessed by 7 different methods sorted in descending order of median values. (c) and (d) shows the corresponding all-atom RMSD for the same analysis.

2.2 Training dataset distributions

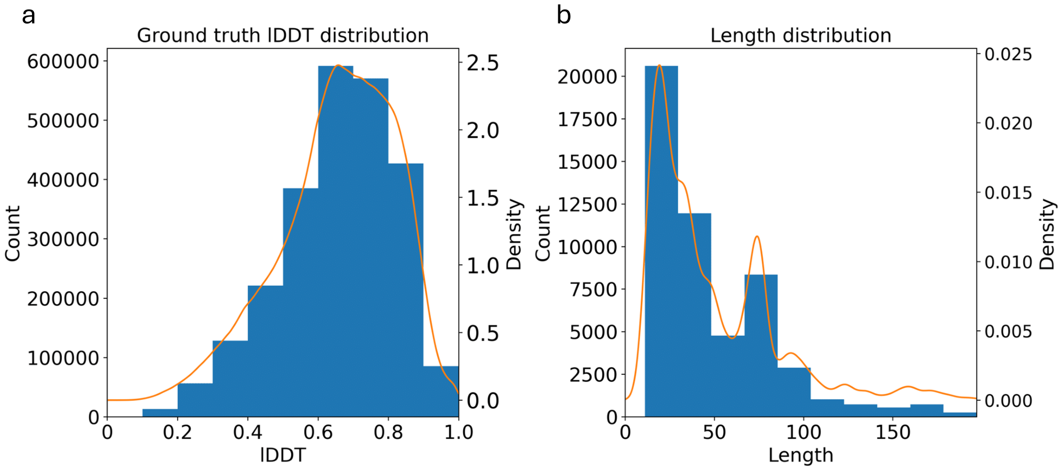

Figure S3: (a) Nucleotide-wise ground truth lDDT distribution of approximately 2.5 million nucleotides on our training set. (b) Sequence length distribution of our training set.

The nucleotide-wise lDDT distribution of the training set, ranging narrowly between 0.4 and 0.8, reflects the relatively accurate predictions of the decoys by various deep learning-based methods. This limited range may lead the model to overestimate the quality of the nucleotide environments.

2.3 Test dataset distributions

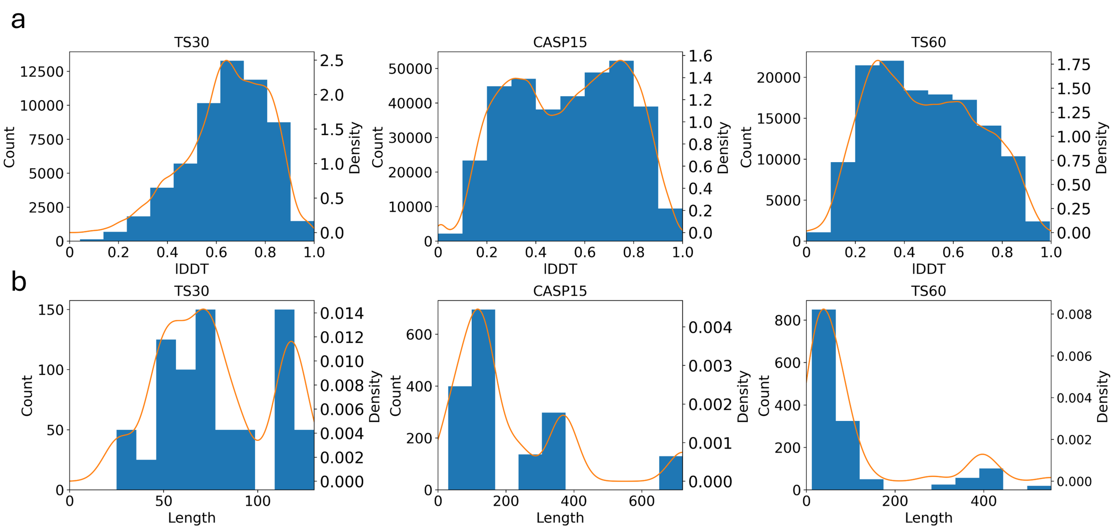

Figure S4: a) Nucleotide-wise ground truth lDDT distributions and b) sequence length distributions for the targets in test and validation sets.

2.4 Interpretation of attention map

We visually analyze the predicted attention weights of the same case study target presented in section 3.3 in the manuscript, i.e., the top-ranked structural model submitted by AIchemy RNA2 (group 232) for the CASP15 target R1108 having a length of 69. Specifically, we plot the attention map extracted from one of the attention heads in the final IPA layer of our trained model. The attention values are normalized using a threshold of 0.2, such that values greater than 0.2 are set to 1, and the rest of the values are set to 0 to facilitate comparison. The attention matrix is an $L \times K$ matrix where L is the sequence length and $K = 20$ represents the K-nearest neighbors selected by lociPARSE as shown in Figure S5. Additionally, we obtain the base-pair annotations from the chosen 3D decoy structure using MC-Annotate [3] and generate a corresponding $L \times K$ matrix by mapping the $L \times L$ outputs to K-nearest neighbor indices of each nucleotide.

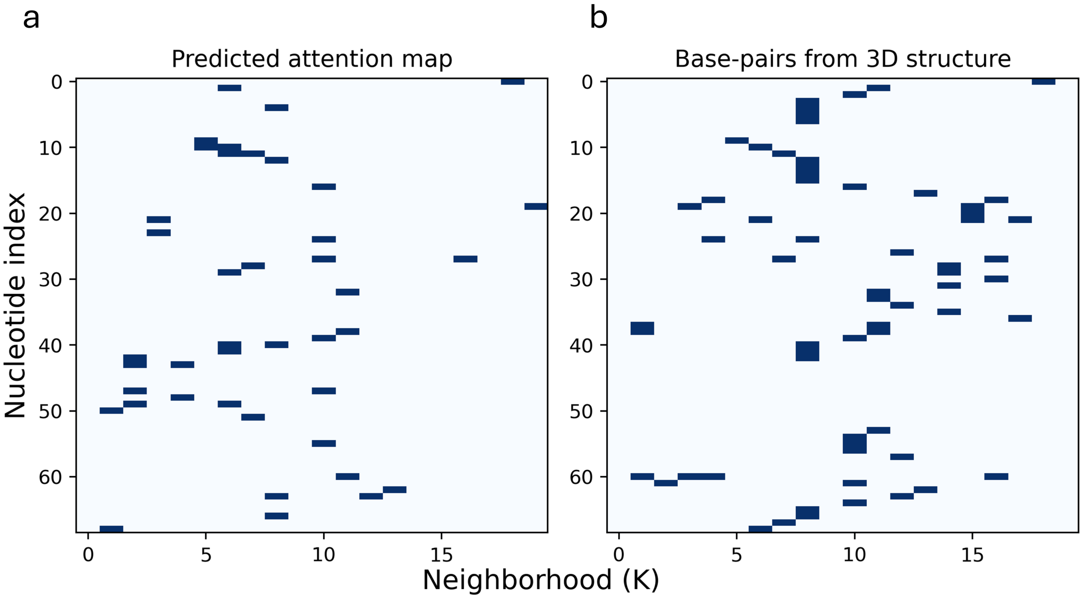

Figure S5: Comparison between a) predicted attention map from the final layer of the model and b) base pairs extracted from the RNA 3D structure. Here, the pixels annotated by dark blue color in both maps correspond to a value of 1, indicating higher attention strength or the presence of a base pair respectively.

A visual comparison between the two maps reveals that the attention weights learned by lociPARSE primarily focus on the base-paired regions of the RNA structure, as indicated by the higher attention weights assigned to those positions, while less attention is given to non-paired nucleotide interactions. Base-pairing interactions are the fundamental building blocks of RNA structure, stabilizing local conformations and directing the overall folding of the RNA molecule [4, 5]. This suggests that the trained model of lociPARSE can implicitly learn the physical principles governing base-pairing interactions, allowing it to capture meaningful structural features consistent with RNA 3D geometry.

2.5 Distinguishability on test sets

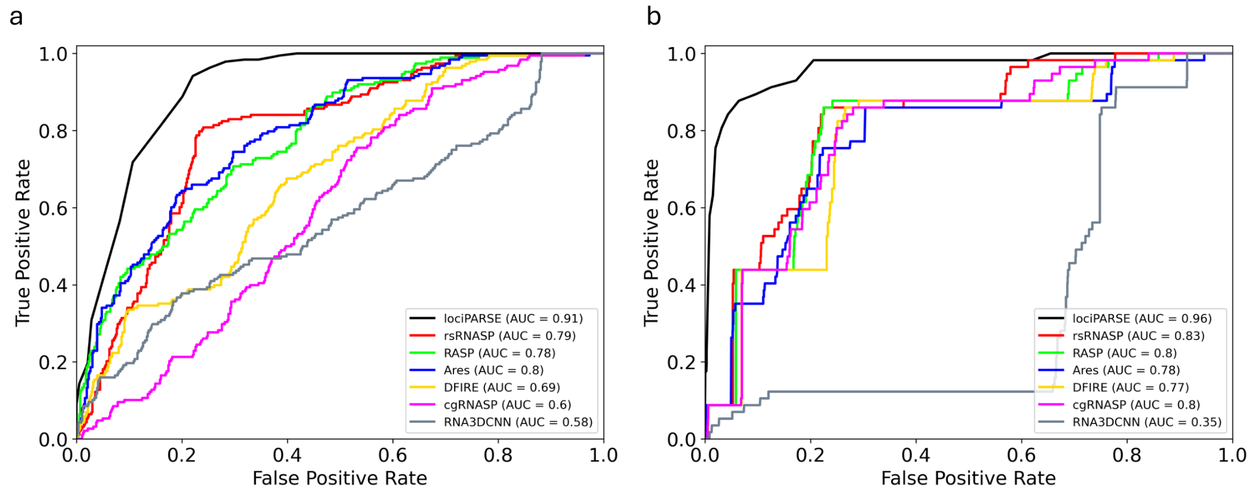

Figure S6: Distinguishability between 'good' and 'bad' models of lociPARSE compared to other methods on a) Test set 30 and b) CASP15 set. Receiver operating characteristic (ROC) curves with the area under the curve (AUC) values reported, where a threshold of lDDT > 0.75 is used to separate 'good' and 'bad' models.

References

[1] Sumit Tarafder and Debswapna Bhattacharya. RNA 3D structural models used to train, test and validate lociPARSE, 2024.

[2] Raphael JL Townshend, Stephan Eismann, Andrew M Watkins, Ramya Rangan, Masha Karelina, Rhiju Das, and Ron O Dror. Geometric deep learning of RNA structure. *Science*, 373(6558):1047–1051, 2021.

[3] Patrick Gendron, Sébastien Lemieux, and François Major. Quantitative analysis of nucleic acid three-dimensional structures. *Journal of molecular biology*, 308(5):919–936, 2001.

[4] Michael Sarver, Craig L Zirbel, Jesse Stombaugh, Ali Mokdad, and Neocles B Leontis. FR3D: finding local and composite recurrent structural motifs in RNA 3D structures. *Journal of mathematical biology*, 56:215–252, 2008.

[5] Tomasz Waleń, Grzegorz Chojnowski, Przemysław Gierski, and Janusz M Bujnicki. ClaRNA: a classifier of contacts in RNA 3D structures based on a comparative analysis of various classification schemes. *Nucleic acids research*, 42(19):e151–e151, 2014.
